# Energy Gradients Structure Microbial Communities Across Sediment Horizons in Deep Marine Sediments of the South China Sea

**DOI:** 10.3389/fmicb.2018.00729

**Published:** 2018-04-11

**Authors:** Michael F. Graw, Grace D'Angelo, Matthew Borchers, Andrew R. Thurber, Joel E. Johnson, Chuanlun Zhang, Haodong Liu, Frederick S. Colwell

**Affiliations:** ^1^College of Earth, Ocean, and Atmospheric Science, Oregon State University, Corvallis, OR, United States; ^2^Department of Microbiology, College of Science, Oregon State University, Corvallis, OR, United States; ^3^Department of Biochemistry and Biophysics, College of Science, Oregon State University, Corvallis, OR, United States; ^4^Department of Earth Sciences, University of New Hampshire, Durham, NH, United States; ^5^State Key Laboratory of Marine Geology, Tongji University, Shanghai, China

**Keywords:** microbial ecology, marine sediment, 16S rRNA, microbial communities, International Ocean Discovery Program

## Abstract

The deep marine subsurface is a heterogeneous environment in which the assembly of microbial communities is thought to be controlled by a combination of organic matter deposition, electron acceptor availability, and sedimentology. However, the relative importance of these factors in structuring microbial communities in marine sediments remains unclear. The South China Sea (SCS) experiences significant variability in sedimentation across the basin and features discrete changes in sedimentology as a result of episodic deposition of turbidites and volcanic ashes within lithogenic clays and siliceous or calcareous ooze deposits throughout the basin's history. Deep subsurface microbial communities were recently sampled by the International Ocean Discovery Program (IODP) at three locations in the SCS with sedimentation rates of 5, 12, and 20 cm per thousand years. Here, we used Illumina sequencing of the 16S ribosomal RNA gene to characterize deep subsurface microbial communities from distinct sediment types at these sites. Communities across all sites were dominated by several poorly characterized taxa implicated in organic matter degradation, including *Atribacteria, Dehalococcoidia*, and *Aerophobetes*. Sulfate-reducing bacteria comprised only 4% of the community across sulfate-bearing sediments from multiple cores and did not change in abundance in sediments from the methanogenic zone at the site with the lowest sedimentation rate. Microbial communities were significantly structured by sediment age and the availability of sulfate as an electron acceptor in pore waters. However, microbial communities demonstrated no partitioning based on the sediment type they inhabited. These results indicate that microbial communities in the SCS are structured by the availability of electron donors and acceptors rather than sedimentological characteristics.

## Introduction

Several non-exclusive theories exist to explain the factors driving the assembly of biological communities. Many current theories are rooted in the concept of patch dynamics (Wu and Loucks, 1995), which considers the roles that ecological disturbance (Rykiel, 1985), community succession (McCook, 1994), and connectivity (Moilanen and Nieminen, 2002) play in the formation of distinct communities across spatial scales relevant to the communities under study. However, the scale of patches, and particularly borders between patches, are increasingly being appreciated as drivers of community composition. For example, ecotones, the transitional region between patches (Smith et al., 1997), increase biodiversity by incorporating multiple patches into the quantification of a single “community.”

Recently, the overarching framework of metacommunity dynamics has been proposed to synthesize the above biogeographic concepts, advancing our understanding of those processes that govern communities over multiple spatial and temporal scales (Leibold et al., 2004). This framework has led to advances in our understanding of community assemblage in a variety of habitats, including riverine systems (Brown et al., 2011), wetlands (Johnson et al., 2013), and the human microbiome (Costello et al., 2012). The deep marine subsurface, that is the deeply buried sediment column below the depth of current interaction with surface oceanographic processes, is an area where patches varying in energetics and biogeochemical history exist on a variety of spatial scales. However, the assembly rules for microbial communities in this environment remain largely unknown (D'Hondt et al., 2004; Teske, 2006).

Microbial communities in the deep marine subsurface exist in a unique environment where gradients in organic matter and electron acceptor availability, dispersal limitation, and energetics are intertwined (Jørgensen and Boetius, 2007; Hoehler and Jørgensen, 2013). Subsurface communities are considered to be largely governed by the niche spaces available to them (O'Malley, 2008). Coupled with observations that microbial biomass and richness decrease exponentially with sediment depth (Kallmeyer et al., 2012) and age (Walsh et al., 2016) as the energy available from organic matter degradation diminishes, this concept has led to an energetics-based view of microbial community structure in the subsurface (LaRowe and Van Cappellen, 2011; Bienhold et al., 2012). The role of dispersal, a pillar of metacommunity dynamics, in microbial communities has only recently been explored through the burgeoning field of microbial biogeography (Martiny et al., 2006). Within marine sediments, microbial dispersal is thought to be limited by both the porosity and permeability of the sediment matrix (Orsi et al., 2013) and the energy available to microorganisms (Hoehler and Jørgensen, 2013).

The abundance of organic matter and the electron acceptor available for its oxidation vary as a function of both sediment depth (time) and seafloor location (space) and are known to influence the metabolic processes, and thus microbial communities, present in the marine subsurface (Jørgensen and Boetius, 2007; Walsh et al., 2016). In marine sediments with moderate to high organic inputs, for example in coastal areas with sedimentation rates of millimeters per year or more, oxygen is entirely consumed within the top millimeters to centimeters of the sediment column and replaced by sulfate as the dominant electron acceptor available to microorganisms for organic matter degradation (Froelich et al., 1979). With increasing depth, the supply of sulfate is depleted and methane is formed from CO_2_ reduction (Whiticar et al., 1986). The result is two broad biogeochemical zones: a shallower sulfate reduction zone and a deeper methanogenic zone. The gradient from sulfate-replete pore water in the sulfate reduction zone to sulfate-depleted, methane-bearing pore water in the methanogenic zone represents a significant shift in energy and niche availability and is frequently associated with changes in microbial community composition (Nunoura et al., 2016; Walsh et al., 2016), including the microbial consortia that drive the anaerobic oxidation of methane (AOM) (Hinrichs et al., 1999; Boetius et al., 2000) at the sulfate-methane transition zone (SMTZ). In addition, Kallmeyer et al. (2012) demonstrated the importance of overlying oceanographic productivity in influencing the abundance of subsurface microorganisms, thus identifying the oceanographic environment as a relevant factor in structuring subsurface communities.

One region that provides a heterogeneous subsurface to enable quantifying the influence of gradients in electron acceptors and donors on subsurface microbial community structure is the South China Sea (SCS). The SCS is an enclosed sea with highly variable sedimentation rates across its several subbasins (Huang and Wang, 2006) and deep sediments from the SCS were recently sampled by the International Ocean Discovery Program (IODP) (Expedition 349 Scientists, 2014). Over the history of the SCS, episodic deposition of turbidites and volcanic ashes from the landmasses surrounding the basin have resulted in lithostratigraphic changes manifested as discrete horizons within the sediment column. Horizons of marine-derived calcareous microfossils compose carbonate oozes that are interspersed in the sediment column as a result of changes in surface paleo-oceanographic conditions (Wang et al., 2000). The variations in sediment depositional patterns across the basin, the variable lithologic sequences in the deep subsurface sediments, and the basin's relative isolation from the global oceans make the SCS well-suited for studying habitat heterogeneity and microbial community assemblage in the deep subsurface. In this study, we ask what factors impact microbial community assemblage in the deep subsurface, focusing on applying the ecological constructs discussed above on spatial and temporal scales appropriate to microbial communities. We hypothesize that microbial community structure in the deep subsurface of the SCS will be determined primarily by selection and dispersal limitations both along gradients in organic matter and electron acceptor availability and across changing sediment characteristics.

## Materials and methods

### Study site and sample collection

Three locations in the SCS were cored during IODP Expedition 349 at sites U1431 [15° 22.5379′ N, 117° 00.0022′ E, 4240 meters below sea level (mbsl)], U1432 (18° 21.0831′ N, 116° 23.4504′ E, 3829 mbsl), and U1433 (12° 55.1380′ N, 115° 02.8345′ E, 4379 mbsl) using an advanced piston core. Cores were split lengthwise onboard to reveal geological interfaces and samples were collected where visible interfaces were observed throughout the cores, usually both from sediments above and below the interface. Exposed sediment was aseptically scraped away prior to sampling and samples were collected only from the center of the cores, avoiding the outside edges of the cores that may have contacted the core barrel. Fifteen samples representing eight interfaces were collected from site U1431, six samples representing three interfaces were collected from site U1432, and 20 samples representing 10 interfaces and two depths at which no interfaces were observed were collected from site U1433. Samples were stored at −80°C until analysis. Down-core measurements of sediment methane concentrations, alkalinity, and pore water sulfate, ammonium, and phosphate were made onboard (Expedition 349 Scientists, 2014). Sediment ages were calculated based on paleomagnetic analysis and nanofossil characterization for each site (Expedition 349 Scientists, 2014).

All samples described in this study were collected using an advanced piston core (APC), a tool that minimizes core disturbance and is designed to recover undisturbed sample material from unconsolidated sediments (Smith et al., 2000; Morono and Inagaki, 2016). Consistent with other deep microbiology coring studies that used a piston coring tool or an APC (Inagaki et al., 2003; Walsh et al., 2016), our study focused on samples that were collected from relatively shallow depths where an APC could be used to penetrate sediments without disruption.

### Sediment composition and texture

#### Smear slide microscopy

Sediment composition and texture was determined post-expedition by examination of the split core photos and sediment subsamples using smear slide microscopy, per standard IODP protocols (e.g., Marsaglia et al., 2013). Using a transmitted light polarizing petrographic microscope, both the grain size and the relative abundance of dominant components in a sample were determined. Sediment names follow the Ocean Drilling Program sediment-classification scheme of Mazzullo et al. (1988). Modifiers to the principal name were determined based on both the abundance and type of the non-principal component or components (e.g., lithogenic or biogenic). The presence of authigenic minerals or other noticeable components such as organic matter or unique trace minerals are also recorded. Because this study targeted discernable lithologic boundaries determined at sea, in this paper we focus on the compositional characteristic rather than the evolution of the sedimentary record through time, which is discussed in detail in Liu et al. (2017).

#### TOC and δ^13^C measurements

We used an *Elementar Vario PYROcube* elemental analyzer and *an IsoPrime VisION* stable isotope mass spectrometer to quantify the amount of total organic carbon (TOC) and its isotopic composition for each sediment type. Prior to TOC analysis, any inorganic carbon (IC) in the form of biogenic, authigenic, and/or detrital CaCO_3_ was dissolved using 6% sulfurous acid applied to weighed samples in amounts and steps optimized for carbonate-rich sediments (Phillips et al., 2011). Isotopic ratios are reported relative to the Vienna Pee Dee Belemnite (%0 VPDB). A replicated internal sample standard (IODP Exp. 353 U1446B 1H3, 130–135 cm) was run repeatedly throughout the sample analysis with a mean and standard deviation for TOC: 1.70 wt. %, s.d. < 0.01; and δ^*13*^*C:* −17.1%0, s.d. 0.2.

### DNA extraction, amplification, and sequencing

DNA was extracted from 0.25 g of wet sediment using a MoBio PowerSoil DNA Isolation Kit. Extracted DNA was amplified in technical triplicate 25 uL reactions using universal 16S rRNA gene primers 515-fwd and 806-rev with Illumina sequencing adapters and barcodes as described previously (Caporaso et al., 2012). Triplicate PCR products were pooled, visualized on an agarose gel, and cleaned using a MoBio UltraClean PCR Clean-Up Kit. PCR products were quantified using a Qubit fluorometer and pooled prior to sequencing. Paired-end 250 bp sequencing was performed on an Illumina MiSeq at Oregon State University's Center for Genome Research and Biocomputing. A sediment-free extraction was amplified and sequenced alongside sediment samples.

#### Sequence processing and statistical analysis

Sequences were processed using mothur (v 1.38.0; Kozich et al., 2013). Reads were clustered at 97% and classified against the SILVA database (v. 119). Singleton OTUs were removed prior to analysis. OTUs that were present in greater than 1% relative abundance in the negative (sediment-free) control and classified as common human or kit contaminants (Salter et al., 2014) were removed from the dataset. To confirm their identity, phylogenetic trees were generated for all OTUs classified as anaerobic methanotrophic archaea (ANME) and for the 8 most abundant OTUs classified to known sulfate-reducing bacteria lineages with Fasttree (v. 2.1.10; Price et al., 2010) using the Jukes-Cantor model (Figures S1, S2).

Diversity metrics (Shannon-Weiner index Magurran, 1988, Pielou's evenness Pielou, 1966), and number of OTUs present) were calculated prior to rarefaction. Samples were rarefied to 4,093 sequences, and the community dissimilarity matrix was computed using weighted UniFrac distances (Lozupone et al., 2006) from both untransformed and square root-transformed OTU tables; results from the former are reported unless square root transformation altered the significance of a statistical test. All statistical analyses were performed using the *vegan* package for R (v. 2.3-3; Oksanen et al., 2013) and the PERMANOVA+ package in PRIMER 6 (Gorley and Clarke, 2006). Distance-based linear modeling (Wasmund et al., 2014) was used to determine the best subsets of measured environmental parameters for predicting microbial community structure, while environmental fitting (Carr et al., 2015) was used to determine the correlation between individual environmental parameters and microbial community structure.

Sequences were deposited to NCBI under accession number PRJNA362622.

## Results and discussion

### Core descriptions, sedimentological characterization, and carbon parameters

**Figure 1:**
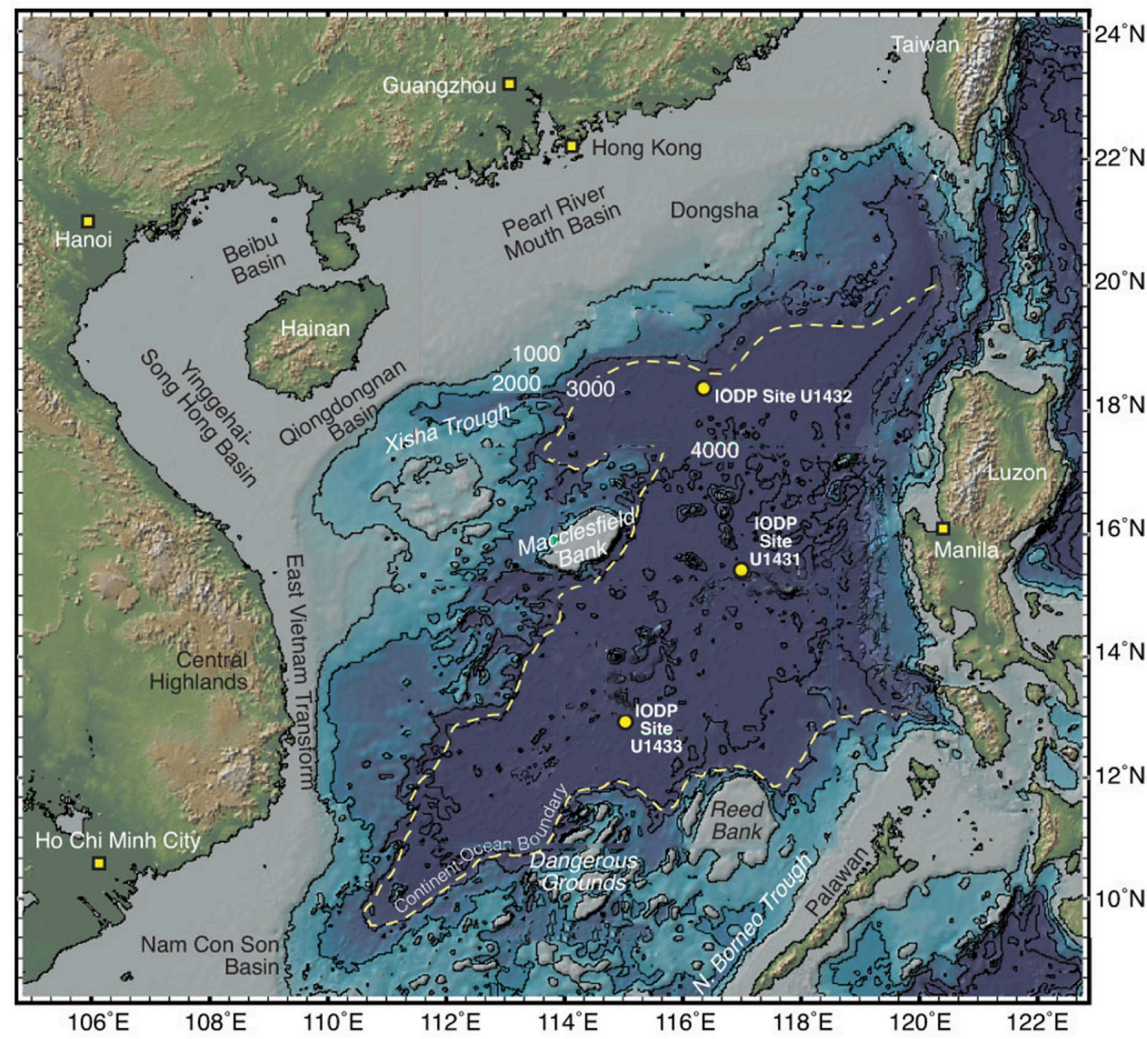
Map of the South China Sea showing the three sites sampled in this study, modified from Expedition 349 Scientists (2014).

**Figure 2:**
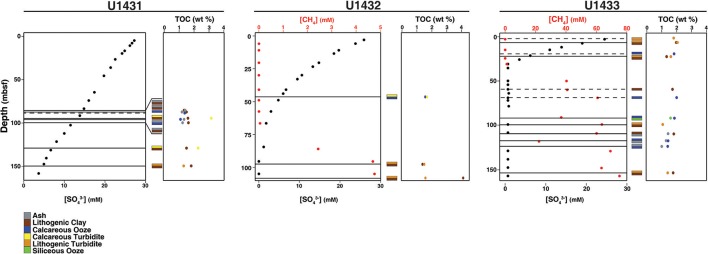
Pore water chemistry, TOC content, and sedimentology of cores in this study. Horizontal lines indicate the sediment depths sampled. Dashed lines indicate that only one sediment horizon, rather than adjacent horizons of different sediment composition, was obtained from that depth.

Sites U1431 and U1433 sampled the abyssal East and Southwest Subbasins, respectively, of the SCS, while site U1432 sampled the base of the southern Chinese continental margin (Figure 1; Table S1). Sedimentation rates were highest at site U1433 (20 cm/kya), moderate at site U1432 (12 cm/kya), and lowest at site U1431 (5 cm/kya) (Expedition 349 Scientists, 2014). Sediments ranged in age from 20 to 900 kya at sites U1432 and U1433, and from 1.7 to 3 mya at site U1431. Sulfate concentrations decreased linearly with depth at site U1431, but no methane was detected at any depth (Figure 2). In sites U1432 and U1433, samples from both the sulfate reduction zone and methanogenic zone were sampled. All samples that we collected were from core sections that were intact and visually undisturbed indicating effective deployment of the APC. Furthermore, porewater measurements of sulfate in whole round cores indicated progressive decrease in this anion and no apparent alteration by drilling fluids that are composed of seawater (Figure 2). We also noted that our samples did not contain microbial taxa that are typical in seawater.

Sedimentological characterization of the samples from smear slides revealed several distinct sediment lithologies: volcanic ashes, lithogenic clays, lithogenic and calcareous turbidites, calcareous ooze, and siliceous ooze (Figure S3). The volcanic ashes were primarily composed of silt-sized glass shards and minor mineral grains and lacking biogenic microfossils. There are two notable exceptions, however: samples U1433A 12H-4 137–139 cm and U1433A 13H-3 87–89 cm contained minor amounts of siliceous microfossil remains (diatoms, radiolarian, and sponge spicules) and both appear to have undergone some chemical alteration (*in situ* weathering) since deposition. Measurable TOC (described below) in all the ashes is likely derived during the descent through the water column or during reworking on the seafloor, but was not visible in smear slide at 630x magnification. The lithogenic clays ranged in grain size and generally were characterized by fine grained mineral and/or lithic content, with no or trace amounts of biogenic shell fragments. All but one of the turbidite occurrences were dominated by lithogenic minerals and/or rock fragments, with minor amounts of biogenic fossil (carbonate or silica) materials. The remaining turbidite (U1432C 6H-1 47–49 cm) contained significant calcareous shell materials from foraminifera and calcareous nannofossils and is considered a calcareous turbidite in this study. Calcareous oozes were characterized by >60% biogenic calcium carbonate in the form of foraminifera and calcareous nannofossil shells and shell remains. Siliceous oozes were characterized by >60% biogenic silica in the form of diatoms, radiolarian, and sponge spicules.

TOC content in the sediments averaged 1.5% by weight and did not differ significantly between sites (ANOVA *df* = 2, 37, *F* = 2.24, *p* = 0.12). However, TOC content differed significantly between sediment lithologies (*df* = 6, 33, *F* = 2.86, *p* = 0.03). TOC content was also significantly higher in the sulfate reduction zone than in the methanogenic zone at sites U1432 and U1433 (Wilcoxon rank-sum *p* = 0.02). Organic carbon C/N in all sediment horizons ranges from 9 to 16 (Table S1), suggestive of a predominantly marine phytoplankton source. Carbon isotopic depletion of the TOC in all sediment horizons, however, ranges from −23 to −26%0 (Table S1), which is more depleted than a pure marine phytoplankton source (−17 to −22%0) (Burdige, 2006) suggestive of some terrestrial, C3 plant material. Given these results, it is likely that all of the samples contain a mixture of both marine and terrestrial organic carbon.

### Geographic patterns of microbial community structure

Illumina sequencing of the 16S rRNA gene yielded 721,945 sequences that were clustered into 5,453 OTUs (97% identity). These OTUs represented 44 bacterial phyla and 3 archaeal phyla (OTU table in Data Sheet 2; taxonomic assignments in Data Sheet 3).

**Figure 3:**
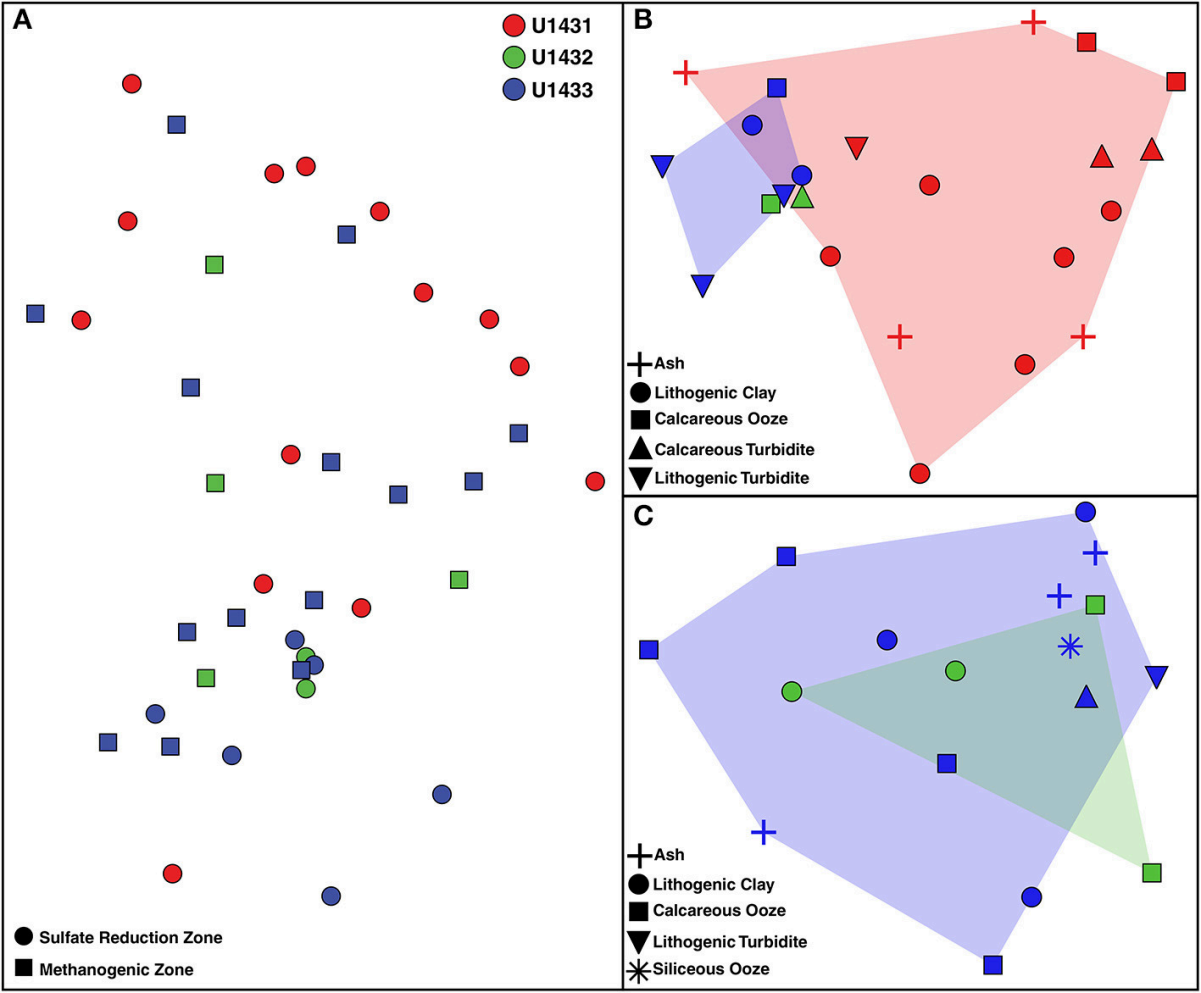
Non-metric multidimensional scaling plot of microbial community composition across **(A)** all communities, **(B)** communities in sulfate reduction zone sediments, and **(C)** communities in methanogenic zone sediments. Two-dimensional stresses of 0.10, 0.08, and 0.07, respectively. Red, green, and blue shading indicates the spread of communities from sites U1431, U1432, and U1433, respectively, in ordination space.

**Figure 4:**
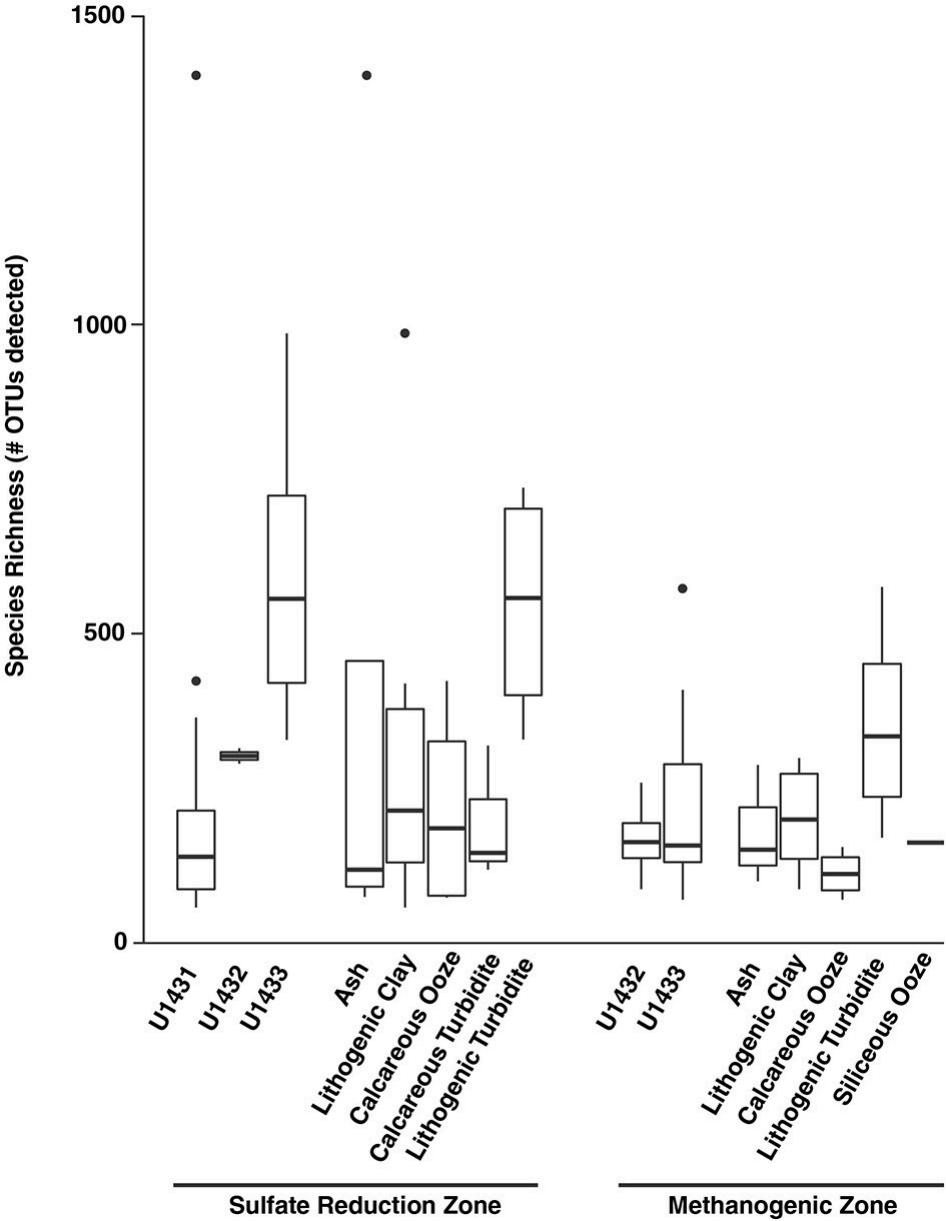
Number of detected species at the OTU level (97% identity) across sites and sediment lithologies for sulfate reduction zone and methanogenic zone communities. Boxes indicate first and third quartiles of data ranges and whiskers extend to highest and lowest points within 1.5 times the interquartile range. Dots indicate outlying points.

Patterns in microbial community structure and diversity were strongly associated with the seafloor location within the SCS that communities inhabited. Microbial community composition was significantly distinct between all sites (PERMANOVA *df* = 2, 38 pseudo-F = 2.08, *p* = 0.03; Figure 3), and pairwise comparison revealed that this was driven largely by compositional differences between communities inhabiting sulfate reduction zone sediments at sites U1431 and U1433 (PERMANOVA *t* = 1.81, *p* = 0.02). Microbial diversity was also significantly higher in site U1433 than U1431 (Shannon-Weiner index, Kruskal-Wallis *df* = 1, 33, *p* = 0.030; Figure 4). Of the 5,453 OTUs detected by sequencing, 2,399 (17 bacterial and 2 archaeal classes) were unique to site U1433, while only 798 and 220 OTUs (4 and 2 bacterial classes) were unique to sites U1431 and U1432, respectively.

### Electron acceptor availability as a control on microbial community structure

The division of the sediment column into a sulfate reduction zone, in which sediment pore water contains sufficient sulfate to serve as an electron acceptor for organic matter degradation, and a deeper methanogenic zone, in which sulfate is depleted and methane is present, is a common feature in anoxic marine sediments. Although cell-specific energy availability may reach an asymptote within the first several meters of the seafloor, methanogenic zone sediments offer a different set of niche spaces relative to sulfate reduction zone sediments due to the lack of sulfate as an electron acceptor and more highly degraded organic carbon. Thus the gradient from sulfate-bearing to methane-bearing sediments is frequently associated with changes in microbial community composition (Nunoura et al., 2016; Walsh et al., 2016).

Among communities in the SCS, the availability of sulfate and the presence of methane were strongly correlated with changes in microbial community composition. Although methanogenic sediments were not sampled at site U1431, at sites U1432 and U1433 communities were significantly different between sulfate reduction zone and methanogenic zone sediments (PERMANOVA *df* = 1, 24, pseudo-F = 4.33, *p* = 0.003). Sediment age and pore water phosphate concentration predicted 34 and 36% of community variance, respectively, among sulfate reduction zone communities across all sites (Table S2). However, both sediment age and phosphate concentration are significantly different among sites (ANOVA *df* = 2, 38, *F* = 112, *p* < 0.001 and *df* = 2, 38, *F* = 18.16, *p* < 0.001, respectively) and are not predictive of changes in community composition within any single site. Among communities inhabiting methanogenic zone sediments, the amount and isotopic depletion of organic carbon were significantly correlated with microbial community variance (Table S2). Within these geochemical zones, the vertical distance in the sediment column between microbial communities did not correlate with changes in community composition. This is consistent with strong energetic selection based on electron acceptor availability rather than sediment age, which has previously been correlated with microbial richness (Walsh et al., 2016), or dispersal throughout an otherwise homogeneous sediment column.

Microbial community diversity also varied across the gradient from sulfate-bearing to methane-bearing sediments. Within sites U1432 and U1433, microbial richness was significantly higher among sulfate reduction zone communities compared to methanogenic zone communities (Wilcoxon rank-sum *p* = 0.001; Figure 4). Species evenness, however, did not vary between geochemical zones (Wilcoxon rank-sum *p* = 0.61). At all sites, microbial richness in the sulfate reduction zone declined exponentially with sediment age (Regression *p* = 0.010, *r*^2^ = 0.24). Within methanogenic zone sediments, microbial diversity was positively related to methane concentration (Regression *p* = 0.020, *r*^2^ = 0.33) and inversely related to TOC content (Regression *p* = 0.03, *r*^2^ = 0.24). In contrast to communities in the sulfate reduction zone, no relationship was found between sediment age and species richness (Regression *p* = 0.75, *r*^2^ = −0.06).

**Figure 5:**
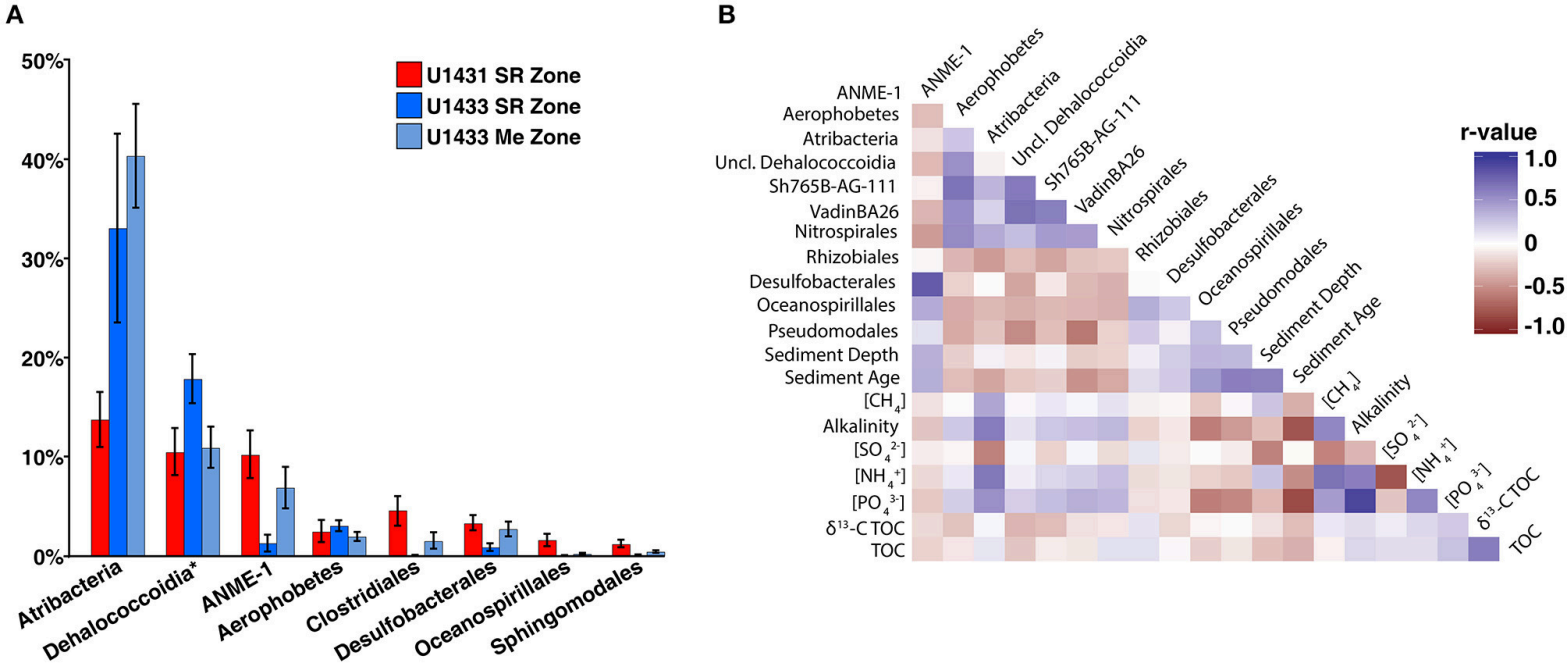
**(A)** Barplot of abundant order-level taxa. Abundant orders were defined as those which contributed greater than 10% of the microbial community in any sample. *Dehalococcoidia* is represented at the class level and includes orders Sh765B-AG-111, VadinBA26, and unclassified *Dehalococcoidia*. **(B)** Heatmap of spearman correlations between abundant order-level taxa and measured sediment geochemical parameters. Only the lower triangular of the heatmap is displayed; heatmap colors indicates the *r*-values of the Spearman correlations.

Several of the most abundant bacterial and archaeal taxa detected in the SCS are frequently associated with either sulfate-replete or methanogenic sediments and their abundances are thought to be largely driven by vertical gradients in electron acceptor availability. Among these are members of the *Atribacteria*, which are commonly associated with methanogenic sediments and are thought to ferment organic matter and provide substrate for methanogens (Carr et al., 2015). In the SCS, however, *Atribacteria* comprised greater than 15% of the community in U1431 and did not show a significant change in abundance between the sulfate reduction and methanogenic zones of sites U1432 and U1433 (Wilcoxon rank-sum *p* = 0.28; Figure 5A). Members of the *Dehalococcoidia*, which are commonly observed in anoxic sediments and mediate reductive dehalogenation and potentially sulfate reduction (Kaster et al., 2014; Wasmund et al., 2014, 2016) were most abundant within sulfate-replete sediments in the SCS. While *Dehaloccoidia* have previously been found to co-occur with *Atribacteria* in methanogenic sediments (Jorgensen et al., 2012), their abundance did not correlate with that of *Atribacteria* (Figure 5B). The abundance of *Atribacteria* was positively correlated with ammonium, methane, phosphate, and TOC concentrations and negatively correlated with sulfate concentration, but the abundance of *Dehalococcoidia* was not predicted by any measured environment variables.

ANME of the ANME-1 clade, which mediate AOM in consortia with sulfate-reducing bacteria (Knittel and Boetius, 2009), made up a significant portion of the microbial community. This was unexpected given that sediments in which both sulfate and methane were present, the geochemical niche at which these organisms are thought to thrive (Knittel and Boetius, 2009; Ruff et al., 2015), were not sampled at any site. Notably, ANME comprised on average 10.5% of the community in U1431, although no methane was detected in pore water from this core. Meanwhile, sulfate reducing bacteria are traditionally thought to be present exclusively in sulfate reduction zone sediments, where they remain competitive by respiring sulfate to degrade organic matter (Muyzer and Stams, 2008). Sulfate-reducing bacteria comprised only 2.5% of the community in U1431 and averaged only 4% of the total community in sulfate-replete sediments across all cores. The abundance of sulfate-reducing bacteria was not significantly different between sulfate-replete and methanogenic sediments at sites U1432 and U1433 (Wilcoxon rank-sum *p* = 0.98). The majority of these sulfate-reducing bacteria were classified as *Desulfobacterales*, a lineage of sulfate-reducing bacteria that have previously been detected in methane seep sediments in the presence of ANME (Roalkvam et al., 2011; Timmers et al., 2015), and their abundance was strongly correlated with that of ANME (Spearman *p* < 0.001, *r*^2^ = 0.62; Figure 5B). Several explanations exist for the observed distribution of sulfate reducing bacteria and ANME away from the current SMTZ. Since the SMTZ can move throughout the sediment column with changes in sedimentation rate and underlying methane fluxes, it is possible that the observed distribution of these organisms reflects variability in the paleo-positions of the SMTZ and represents undegraded DNA from dead or dormant cells (Peketi et al., 2012). However, DNA turnover in marine sediments occurs on timescales of several months (Corinaldesi et al., 2008) and changes in the depth of the SMTZ of greater than 100 m over those timescales are highly unlikely. Alternatively, AOM mediated within the methanogenic zone by sulfate reducing bacteria and ANME has previously been observed and linked to sulfur cycling below the SMTZ (Holmkvist et al., 2011; Treude et al., 2014). Pyrite and barite have been proposed as sources of sulfate in methanogenic sediments (Berner, 1970; Treude et al., 2014). Whether either or both of these sources could produce enough sulfate to support the observed abundances of sulfate reducing bacteria and ANME in methanogenic zone sediments is unclear, since neither the iron nor barite content of the sediments was measured. Within the sulfate reduction zone, it is possible that active AOM is consuming pore water methane to below detectable levels; however, 16S rRNA gene surveys do not provide insight into the metabolic activity of these cells. Lastly, several lines of evidence suggest that ANME may function as methanogens under the thermodynamic conditions of methanogenic zone sediments (House et al., 2009; Lloyd et al., 2011). If this is the case, ANME may account for the observed sediment methane concentrations given that traditional methanogenic lineages were detected only in extremely low abundances in this study. Co-occurring sulfate reducing bacteria could be gaining energy via fermentation of a subset of the organic matter pool and provide substrates to ANME for methanogenesis in the process.

### Role of sedimentological transitions in community structure

The sediment column of the SCS features numerous lithostratigraphic horizons at which sediments of different composition and texture are deposited on top of one another. Despite variations in the sedimentological characteristics of these different sediment types, microbial communities inhabiting SCS sediments were not significantly conserved within a single sediment type nor significantly different across varying sediment types (PERMANOVA *df* = 6, 34, pseudo-F = 1.39, *p* = 0.10). This was found to be the case across all sites and regardless of geochemical zonation (sulfate reduction zone: PERMANOVA *df* = 5, 17, pseudo-F = 1.23, *p* = 0.24; methanogenic zone: PERMANOVA *df* = 4, 13, pseudo-F = 0.16, *p* = 0.20). Considering broader classifications of sediment lithologies, for example grouping all turbidites, did not alter this result (PERMANOVA *df* = 4, 36, pseudo-F = 1.13, *p* = 0.32). Furthermore, no correlation was observed between community similarity and the vertical distance between communities within the sediment column regardless of the site or geochemical zone examined (Mantel test; U1431 *r* = −0.14, *p* = 0.86; U1433 sulfate reduction zone *r* = 0.42, *p* = 0.07; U1433 methanogenic zone *r* = 0.02, *p* = 0.42). These results contrast a previous study of microbial communities in the Sea of Okhotsk, a marginal sea similar to the SCS, which found that communities inhabiting layered ash and clay sediments in a single core were distinct between sediment types (Inagaki et al., 2003). However, several key differences between the sediment environment in the Sea of Okhotsk and the SCS may explain the discrepancy in whether sedimentology is related to microbial community structure. The site sampled in the Sea of Okhostsk was coastal, with a relatively shallow water column (1,225 m) and a high sedimentation rate (100 cm/kya). Many of the lineages identified as key drivers of the community distinction between ash and clay layers in that study, in particular the archaeal phyla *Bathyarchaeota* and *Lokiarchaeota*, were not detected in the SCS. While this could be related to differences in the primers used in that study, these phyla are thought to prefer eutrophic coastal sediments rather than oligotrophic abyssal sediments (Durbin and Teske, 2012) and may indicate a broader divergence in the relationship between sedimentology and microbial community structure between these environments.

In addition, as a result of the complex paleoceanographic history of the SCS, it is likely that the conditions under which the sediments examined in this study were deposited varied greatly (Expedition 349 Scientists, 2014). This may have impacted the content of turbidites and calcareous oozes both prior to deposition and during early diagenesis, resulting in divergent processes affecting sediments of the same origin deposited during different eras. In the case of ashes, alteration in the water column under different oceanographic conditions may have also played a role in reducing the physio-chemical homogeneity of ash deposits.

Interestingly, the degree to which microbial communities inhabiting adjacent sediment horizons diverged was related to the geochemical zone in which the sediments were found. Within sulfate-replete sediments, communities in contacting sediment horizons were significantly similar to each other and distinct from communities from other sulfate reduction zone communities (PERMANOVA *df* = 12, 10, pseudo-F = 2.57, *p* = 0.001). Within methanogenic sediments, however, communities in adjacent sediment horizons were not significantly similar to one another compared to other methanogenic zone communities (PERMANOVA *df* = 7, 10, pseudo-F = 0.94, *p* = 0.58). This finding was confirmed by examining the calculated dissimilarity between adjacent communities in the sulfate reduction and methanogenic zones in sites U1432 and U1433. The average dissimilarity between communities from adjacent sediment horizons was higher among communities in the methanogenic zone than among those in the sulfate reduction zone (Wilcoxon rank-sum *p* = 0.023; Figure S4). Furthermore, the dissimilarity between adjacent communities was unrelated to the sediment types they inhabited (ANOVA *df* = 6, 11, *F* = 0.53, *p* = 0.77). In addition, neither the difference in TOC content nor isotopic depletion between interfacing sediments lithologies was correlated with the dissimilarity between adjacent communities regardless of geochemical zone (Regression; sulfate reduction zone: TOC *r*^2^ = −0.11, *p* = 0.72; δ^13^-C TOC *r*^2^ = −0.05, *p* = 0.48; methanogenic zone: TOC *r*^2^ = 0.30, *p* = 0.12; δ^13^-C TOC *r*^2^ = −0.18, *p* = 0.80). The broad applicability of this finding remains to be seen given the limited number of adjacent communities that were sampled from cores that penetrated both the sulfate reduction zone and the methanogenic zone, the limited number of sites examined for this relationship, and the fact that several types of sedimentological interfaces were only sampled once in this study.

### Biogeography across the South China Sea

Microbial communities inhabiting seemingly homogeneous sediments within marginal seas have previously been found to be conserved across sites within the same basin yet distinct between basins (Liu et al., 2015; Keuter and Rinkevich, 2016). In contrast, microbial communities in the SCS varied significantly between sampled sites in both diversity and composition. Only 37% of detected OTUs were found at all sites, a compositional variance similar to that found between microbial communities inhabiting methane seeps and surrounding non-seep sediments (Pop Ristova et al., 2015). The community variance between sites was largely attributed to differing abundances of members of the phyla *Atribacteria, Chloroflexi, Nitrospira*, and *Aerophobetes* between sites U1432 and U1433 and site U1431. All of these taxa have previously been implicated in organic matter degradation in marine sediments, suggesting that differences in the availability or reactivity of organic matter across the basin may play a role in structuring the biogeography of the SCS.

Sedimentation rate may serve as a proxy for the relative availability of organic matter in marine sediments. Sedimentation rate has previously been demonstrated to exert a primary control on microbial biomass and its decay with depth in marine sediments globally (Kallmeyer et al., 2012). While TOC content was constant across sites sampled in the SCS, sedimentation rate is often considered a better indicator of the relative quantity of organic matter delivered to the seafloor since measured bulk sediment TOC does not reflect the amount of organic carbon remineralized by microorganisms (Tyson, 2001). Sedimentation rate, meanwhile, varied strongly across the SCS from 5 cm/kya at site U1431 to 12 cm/kya at site U1432 and 20 cm/kya at site U1433. Increased organic matter delivery to the seafloor at the latter would increase the total energy available to heterotrophic microorganisms in these sediments relative to site U1431, in turn enabling them to dominate the communities as observed.

Sediment age may also reflect the energetic potential of the sediment column, since microorganisms preferentially oxidize labile over recalcitrant organic matter in recently deposited sediments (Westrich and Berner, 1984). Aged sediments containing a larger recalcitrant fraction may have less energy available via organic matter degradation, thus limiting metabolic niche space (Evans et al., 2005) and the ability of heterotrophic microorganisms to co-exist (Srivastava and Lawton, 1998; Bienhold et al., 2012). Accordingly, sediment age has previously been correlated with decreasing microbial richness (Walsh et al., 2016) and the same relationship was observed among communities in the SCS. In addition, the relative abundances of taxa known to be involved in organic matter degradation in marine sediments, such as *Atribacteria, Aerophobetes, Dehalococcoidia*, and *Nitrospira*, were negatively correlated with sediment age across the SCS (Figure 5B). Thus sediment age, in conjunction with sedimentation rate, may play an important role in structuring microbial communities across sites within the SCS.

### Ecological implications of microbial community structure in the SCS

Ecological models of community assembly have not previously been applied to microbial communities in the deep marine subsurface. Microorganisms in this environment are thought to be dispersal-limited due to the presence of a sediment matrix as well as energetic limitations. Communities in vertically adjacent sediment horizons were more similar in sulfate reduction zone sediments than in methanogenic zone sediments, indicating a potential role for dispersal under moderately favorable energetic conditions. However, this is counterbalanced by the finding that changes in community composition were not correlated with the vertical distance between communities, which suggests that dispersal is not a limiting factor for microbial community distribution within the sediment column or that the community is under such a high degree of connectivity that vertical patterns are lost. Disturbance, which has been found to play a significant role in structuring macrofaunal communities (Supp and Ernest, 2014), did not appear to structure deep marine sediments even though the sampled lithostratigraphic transitions reflect previously disturbed environments. Microbial communities were not significantly different between turbidite, ash, and clay horizons, despite the large-scale physical and geochemical disturbances that ash and turbidite deposition potentially brings to seafloor microbial communities. The concept of ecotones (Smith et al., 1997) also did not explain microbial community assemblage in the SCS, since for ecotones to exist at these sedimentological interfaces the different sediment types must have distinct communities. Successional changes in community structure, driven by changes in electron acceptor availability and sediment age with increasing burial depth, appeared to dominate community assemblage within these sediments (McCook, 1994). In addition, community composition was significantly different between the three sites, potentially reflecting differences in sedimentation rates and labile organic carbon availability across the SCS. These results suggest that within the deep marine subsurface an ecological model incorporating electron acceptor and sedimentation rate or sediment age is the most suitable for predicting microbial community assemblage.

## Author contributions

MG, AT, CZ, HL, and FC contributed to study conception and design. GD and MB performed DNA extraction and amplification. JJ performed sedimentological characterization and TOC measurements. MG, GD, MB, and AT performed statistical analysis. MG, AT, JJ, HL, and FC contributed sections to the manuscript. All authors contributed to manuscript revision and read and approved the submitted version.

### Conflict of interest statement

The authors declare that the research was conducted in the absence of any commercial or financial relationships that could be construed as a potential conflict of interest.
