# Supplementary material for: Energy Gradients Structure Microbial Communities Across Sediment Horizons in Deep Marine Sediments of the South China Sea
Source: Front Microbiol. 2018 Apr 11;9:729. doi: 10.3389/fmicb.2018.00729 (PMC5905238; doi:10.3389/fmicb.2018.00729)

**TABLE S1.** Samples included in this study and associated sedimentological, geochemical, and microbiological attributes. Mbsf, meters below seafloor; δ^13^C-TOC, isotopic depletion of total organic carbon; C/N, sediment carbon to nitrogen ratio.

| **Site** | **Depth (mbsf)** | **Sample name** | **Sediment Age (kya)** | **Lithology** | **[SO_4_^2-^] (mM)** | **[CH_4_] (uM)** | **[NH_4_^+^] (uM)** | **[PO_4_^3-^] (mM)** | **TOC (wt %)** | **δ^13^C-TOC** | **C/N** | **Microbial Diversity (Shannon-Weiner Index)** | **Microbial Evenness (Pielou’s Index)** | **Microbial Richness (# OTUs Detected)** |
| --- | --- | --- | --- | --- | --- | --- | --- | --- | --- | --- | --- | --- | --- | --- |
| U1431 | 85.64 | U1431D 10H6 6-8cm | 1712.8 | Ash | 14.84 | 3.43 | b.d. | 4.21 | 1.402 | -25.880 | 15.07 | 3.44 | 0.75 | 96 |
| U1431 | 85.71 | U1431D 10H6 13-15cm | 1714.2 | Lithogenic Clay | 14.84 | 3.43 | b.d. | 4.21 | 1.330 | -24.569 | 10.14 | 3.52 | 0.68 | 184 |
| U1431 | 87.51 | U1431D 10H7 43-45 | 1750.2 | Ash | 14.84 | 3.43 | b.d. | 4.21 | 1.188 | -26.117 | 11.24 | 5.85 | 0.81 | 1402 |
| U1431 | 87.56 | U1431D 10H7 48-50cm | 1751.2 | Lithogenic Clay | 14.84 | 3.43 | b.d. | 4.21 | 1.489 | -25.557 | 11.01 | 2.42 | 0.60 | 57 |
| U1431 | 87.93 | U1431D 10H8 17-19cm | 1758.6 | Calcareous Ooze | 14.84 | 3.43 | b.d. | 4.21 | 1.370 | -25.358 | 11.56 | 3.24 | 0.76 | 73 |
| U1431 | 94.79 | U1431D 11H5 9-11cm | 1895.8 | Calcareous Turbidite | 13.80 | 3.81 | 5.30 | 0.94 | - | - | - | 4.34 | 0.87 | 145 |
| U1431 | 94.85 | U1431D 11H5 15-17cm | 1897.0 | Lithogenic Clay | 13.80 | 3.81 | 5.30 | 0.94 | 1.577 | -25.072 | 9.31 | 4.49 | 0.91 | 139 |
| U1431 | 95.72 | U1431D 11H5 102-104cm | 1914.4 | Ash | 13.80 | 3.81 | 5.30 | 0.94 | 1.332 | -25.231 | 12.88 | 3.61 | 0.84 | 74 |
| U1431 | 95.77 | U1431D 11H5 107-109cm | 1915.4 | Calcareous Ooze | 13.80 | 3.81 | 5.30 | 0.94 | 1.095 | -24.933 | 13.59 | 3.44 | 0.79 | 77 |
| U1431 | 99.79 | U1431D 12H2 9-11cm | 1995.8 | Ash | 11.52 | 3.1 | 9.90 | 0.54 | 1.231 | -25.579 | 13.36 | 4.04 | 0.82 | 140 |
| U1431 | 99.85 | U1431D 12H2 15-17cm | 1997.0 | Lithogenic Clay | 11.52 | 3.1 | 9.90 | 0.54 | 1.629 | -25.699 | 9.71 | 4.05 | 0.69 | 364 |
| U1431 | 128.77 | U1431D 15H3 27-29cm | 2575.4 | Calcareous Turbidite | 6.65 | 2.22 | 252.9 | 1.19 | - | - | - | 4.23 | 0.89 | 118 |
| U1431 | 128.85 | U1431D 15H3 35-37cm | 2577.0 | Lithogenic Clay | 6.65 | 2.22 | 252.9 | 1.19 | 1.492 | -23.670 | 9.76 | 4.00 | 0.86 | 102 |
| U1431 | 148.97 | U1431D 17H4 132-134cm | 2979.4 | Lithogenic Turbidite | 4.96 | 2.43 | 452.5 | 1.58 | 1.302 | -25.348 | 16.55 | 4.63 | 0.77 | 423 |
| U1431 | 149.03 | U1431D 17H4 138-140cm | 2980.6 | Lithogenic Clay | 4.96 | 2.43 | 452.5 | 1.58 | 1.812 | -25.251 | 11.13 | 3.95 | 0.72 | 243 |
| U1432 | 46.37 | U1432C 6H1 47-49cm | 386.4 | Calcareous Turbidite | 19.75 | 2.51 | 38.00 | 5.99 | 1.680 | -25.286 | 9.01 | 3.94 | 0.68 | 318 |
| U1432 | 46.42 | U1432C 6H1 52-54cm | 386.8 | Calcareous Ooze | 19.75 | 2.51 | 38.00 | 5.99 | 1.572 | -24.552 | 13.46 | 3.76 | 0.66 | 293 |
| U1432 | 97.40 | U1432C 11H5 40-42cm | 811.7 | Lithogenic Turbidite | 0.00 | 4681.3 | 1846.9 | 18.18 | 1.488 | -25.386 | 10.97 | 3.71 | 0.72 | 173 |
| U1432 | 97.45 | U1432C 11H5 45-47cm | 812.1 | Lithogenic Clay | 0.00 | 4681.3 | 1846.9 | 18.18 | 1.378 | -24.814 | 8.74 | 3.23 | 0.72 | 88 |
| U1432 | 108.43 | U1432C 12H6 43-44 | 903.6 | Lithogenic Turbidite | 0.00 | 4748.75 | 1950.3 | 17.64 | 1.517 | -25.465 | 11.09 | 3.95 | 0.71 | 260 |
| U1432 | 108.46 | U1432C 12H6 46- 47cm | 903.8 | Lithogenic Clay | 0.00 | 4748.75 | 1950.3 | 17.64 | 1.574 | -25.116 | 9.76 | 4.21 | 0.83 | 155 |
| U1433 | 4.05 | U1433A 1H3 105-107cm | 20.3 | Lithogenic Turbidite | 24.54 | 5.65 | 573.16 | 41.33 | 1.783 | -23.842 | 9.36 | 5.29 | 0.80 | 734 |
| U1433 | 7.01 | U1433A 1H5 101-103cm | 35.1 | Lithogenic Turbidite | 19.04 | 5.65 | 766.30 | 54.47 | 2.125 | -23.917 | 11.99 | 4.43 | 0.68 | 689 |
| U1433 | 7.08 | U1433A 1H5 108-110cm | 35.4 | Lithogenic Clay | 19.04 | 5.65 | 766.30 | 54.47 | 2.071 | -24.505 | 11.96 | 4.03 | 0.58 | 985 |
| U1433 | 19.35 | U1433A 3H1 95-97cm | 96.8 | Calcareous Ooze | 6.19 | 3.35 | 1716.51 | 73.44 | 1.811 | -23.950 | 11.05 | 2.58 | 0.43 | 423 |
| U1433 | 21.79 | U1433A 3H3 39-41cm | 109.0 | Lithogenic Turbidite | 6.19 | 3.35 | 1716.51 | 73.44 | 1.604 | -25.086 | 12.40 | 3.69 | 0.64 | 328 |
| U1433 | 21.85 | U1433A 3H3 45-47cm | 109.3 | Lithogenic Clay | 6.19 | 3.35 | 1716.51 | 73.44 | 1.430 | -25.329 | 10.55 | 2.26 | 0.37 | 419 |
| U1433 | 59.26 | U1433A 7H2 136-138cm | 296.3 | Lithogenic Clay | 0.73 | 40827.85 | 3011.38 | 37.61 | 1.685 | -23.927 | 11.81 | 1.97 | 0.36 | 246 |
| U1433 | 68.76 | U1433A 8H2 136-138cm | 343.8 | Calcareous Ooze | 0.69 | 60743.7 | 3238.39 | 19.77 | - | - | - | 3.07 | 0.72 | 71 |
| U1433 | 92.00 | U1433A 10H6 93-95cm | 460.0 | Calcareous Ooze | 0.75 | 36868.38 | 3607.75 | 19.77 | 1.812 | -23.590 | 13.89 | 2.56 | 0.51 | 156 |
| U1433 | 92.04 | U1433A 10H6 97-99cm | 460.2 | Siliceous Ooze | 0.75 | 36868.38 | 3607.75 | 19.77 | 1.582 | -25.473 | 12.64 | 1.97 | 0.39 | 161 |
| U1433 | 99.69 | U1433A 11H4 71-73cm | 498.5 | Lithogenic Turbidite | 0.67 | 63571.43 | 3337.03 | 25.25 | 1.107 | -25.524 | 10.65 | 4.55 | 0.72 | 574 |
| U1433 | 99.74 | U1433A 11H4 76-78cm | 498.7 | Lithogenic Clay | 0.67 | 63571.43 | 3337.03 | 25.25 | - | - | - | 2.98 | 0.52 | 300 |
| U1433 | 109.55 | U1433A 12H4 130-132cm | 547.8 | Lithogenic Clay | 0.68 | 60068.64 | 3587.19 | 17.28 | 1.773 | -24.955 | 9.89 | 2.22 | 0.46 | 131 |
| U1433 | 109.62 | U1433A 12H4 137-139cm | 548.1 | Ash | 0.68 | 60068.64 | 3587.19 | 17.28 | 1.457 | -24.318 | 10.93 | 3.18 | 0.63 | 155 |
| U1433 | 117.06 | U1433A 13H3 82-84cm | 585.3 | Calcareous Ooze | 0.68 | 22236.2 | 3587.19 | 17.28 | 1.468 | -24.387 | 12.05 | 1.72 | 0.38 | 91 |
| U1433 | 117.11 | U1433A 13H3 87-89cm | 585.6 | Ash | 0.68 | 22236.2 | 3587.19 | 17.28 | 1.354 | -25.846 | 9.11 | 4.01 | 0.71 | 292 |
| U1433 | 124.13 | U1433A 14H1 123-125cm | 620.7 | Ash | 0.69 | 69198.71 | 3206.20 | 9.51 | 1.004 | -26.157 | 13.28 | 3.53 | 0.76 | 104 |
| U1433 | 124.18 | U1433A 14H1 128-130cm | 620.9 | Calcareous Ooze | 0.69 | 69198.71 | 3206.20 | 9.51 | 1.408 | -24.491 | 10.54 | 3.31 | 0.68 | 134 |
| U1433 | 153.57 | U1433A 17H2 71-73cm | 767.9 | Lithogenic Turbidite | 6.19 | 75147.39 | 1716.51 | 73.44 | 1.411 | -24.805 | 13.25 | 4.32 | 0.72 | 410 |
| U1433 | 153.63 | U1433A 17H2 77-79cm | 768.2 | Lithogenic Clay | 6.19 | 75147.39 | 1716.51 | 73.44 | 1.699 | -24.560 | 10.25 | 2.95 | 0.52 | 284 |

**TABLE S2.** Results of fitting sedimentological and geochemical data to microbial community composition across subsets of samples. Variables in left-most column refer to the subset of variables that best predict microbial community structure by distance-based linear modeling. The best model was determined by model r^2^ values, and the r^2^ value of the best model is given in the left-most column. R^2^ and p values within the table refer to the results of fitting single environmental variables to the community dissimilarity observed for the given subset of samples. Sites U1432 and U1433 were analyzed together since they hosted similar microbial communities and both sampled the sulfate reduction and methanogenic zones. δ^13^C-TOC, isotopic depletion of total organic carbon.

|  | **Sediment Depth** | **Sediment Age** | **TOC** | **δ^13^C-TOC** | **[SO_4_^2-^]** | **[CH_4_]** | **Alkalinity** | **[NH_4_^+^]** | **[PO_4_^3-^]** |
| --- | --- | --- | --- | --- | --- | --- | --- | --- | --- |
| **All Samples**  Depth, δ^13^-C TOC  r^2^ = 0.18 | **r^2^ = 0.23**  **p = 0.01** | **r^2^ = 0.27**  **p = 0.002** | r^2^ = 0.16  p = 0.06 | r^2^ = 0.08  p = 0.23 | r^2^ = 0.05  p = 0.36 | r^2^ = 0.00  p = 0.96 | r^2^ = 0.12  p = 0.08 | r^2^ = 0.07  p = 0.23 | **r^2^ = 0.23**  **p = 0.01** |
| **U1431**  Alkalinity  r^2^ = 0.09 | r^2^ = 0.02  p = 0.89 | r^2^ = 0.002  p = 0.99 | r^2^ = 0.26  p = 0.22 | r^2^ = 0.21  p = 0.30 | r^2^ = 0.04  p = 0.74 | r^2^ = 0.12  p = 0.46 | r^2^ = 0.16  p = 0.37 | r^2^ = 0.03  p = 0.81 | r^2^ = 0.06  p = 0.68 |
| **U1432/U1433**  Depth, δ^13^-C TOC  r^2^ = 0.26 | **r^2^ = 0.31**  **p = 0.02** | **r^2^ = 0.24**  **p = 0.04** | **r^2^ = 0.39**  **p = 0.01** | **r^2^ = 0.55**  **p = 0.001** | **r^2^ = 0.41**  **p = 0.004** | r^2^ = 0.21  p = 0.08 | r^2^ = 0.05  p = 0.58 | **r^2^ = 0.35**  **p = 0.01** | r^2^ = 0.17  p = 0.12 |
| **Sulfate reduction zone**  Age  r^2^ = 0.22 | **r^2^ = 0.43**  **p = 0.004** | **r^2^ = 0.34**  **p = 0.01** | r^2^ = 0.12  p = 0.30 | r^2^ = 0.04  p = 0.68 | r^2^ = 0.07  p = 0.51 | n/a | r^2^ = 0.24  p = 0.07 | r^2^ = 0.23  p = 0.07 | **r^2^ = 0.36**  **p = 0.02** |
| **Methanogenic zone**  δ^13^-C TOC  r^2^ = 0.12 | r^2^ = 0.16  p = 0.27 | r^2^ = 0.20  p = 0.20 | **r^2^ = 0.44**  **p = 0.03** | **r^2^ = 0.59**  **p = 0.01** | n/a | r^2^ = 0.12  p = 0.41 | r^2^ = 0.09  p = 0.50 | r^2^ = 0.12  p = 0.42 | r^2^ = 0.09  p = 0.50 |

**Figure S1.** Phylogenetic tree of all OTUs classified to anaerobic methanotrophic lineages and selected representative sequences. Sequences from this study are in bold. Confidence values are based on 1,000 resamplings.

**
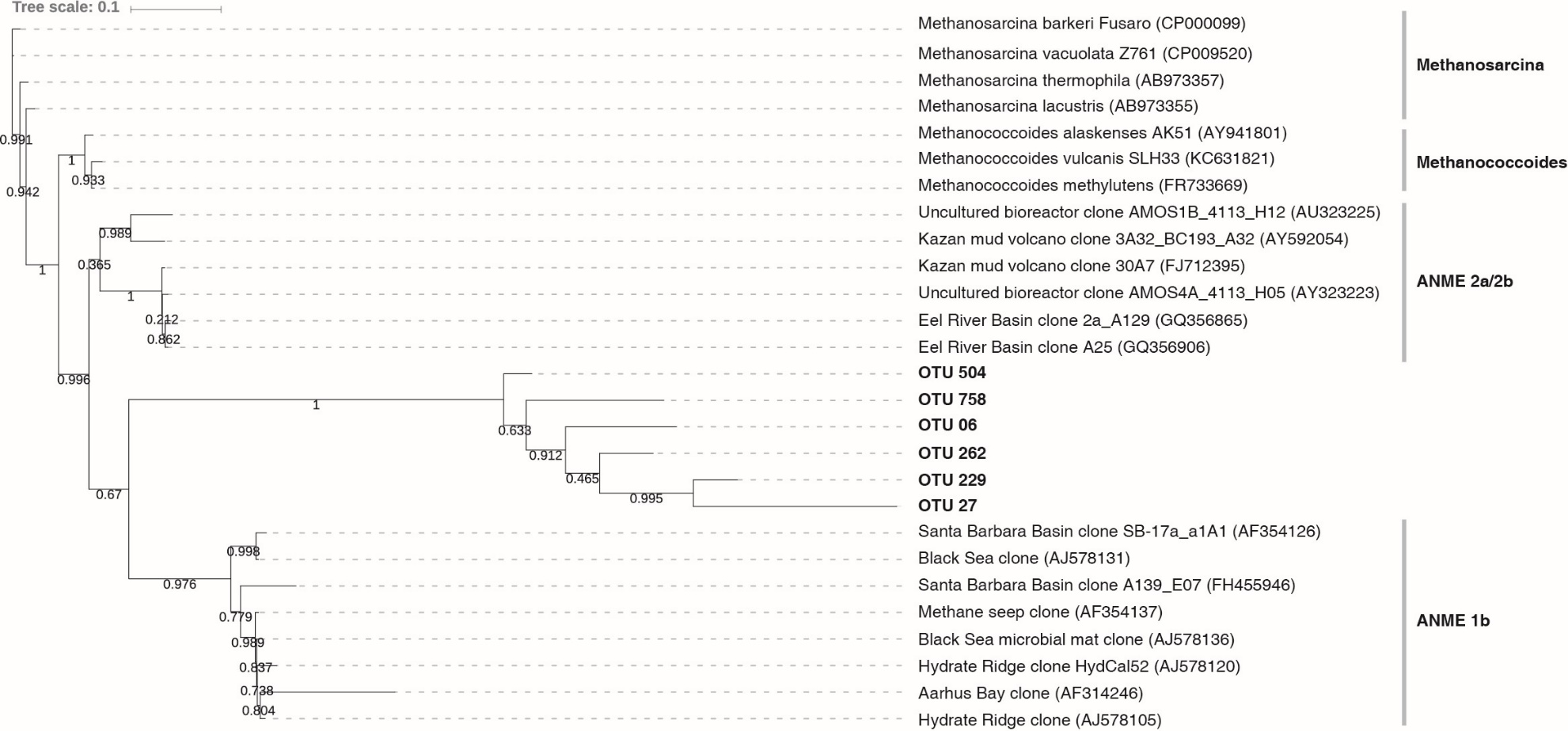
**

**Figure S2.** Phylogenetic tree of the 8 most abundant OTUs classified to known sulfate reducing bacteria lineages and selected representative sequences. Sequences from this study are in bold. Confidence values are based on 1,000 resamplings.

**
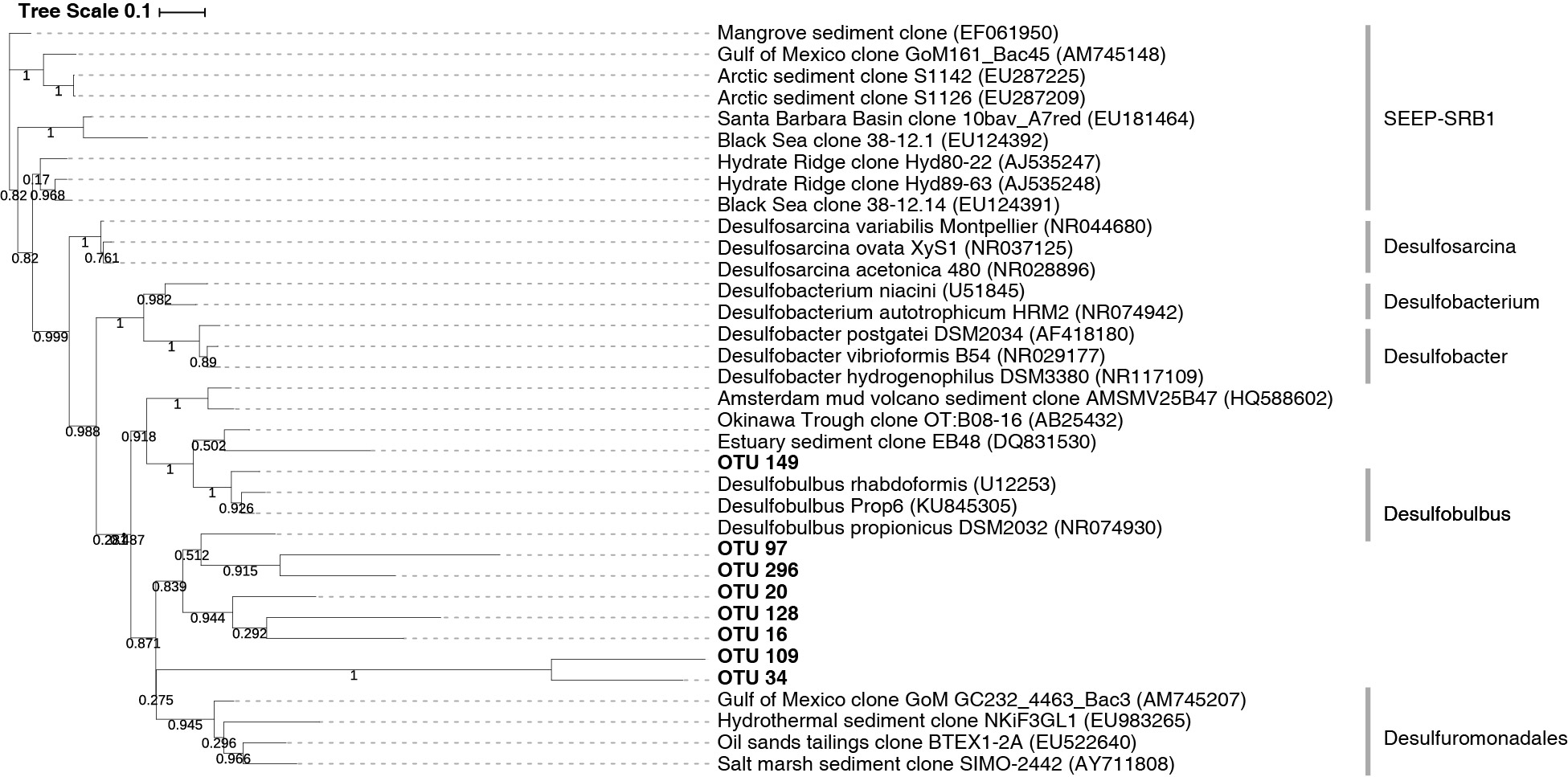
**

**Figure S3.** Smear slide photomicrographs showing typical sediment characteristics for (a) volcanic ash in plane polarized light, note angular glass shards; (b) calcareous ooze in cross polarized light, note the dominance of calcareous nannofossils; (c) siliceous ooze in plain polarized light, note the dominance of siliceous microfossil fragments; (d) lithogenic clay in plane polarized light, note the lack of biogenic particles; and (e) lithogenic silt turbidite in cross polarized light, note the dominance of mineral grains.


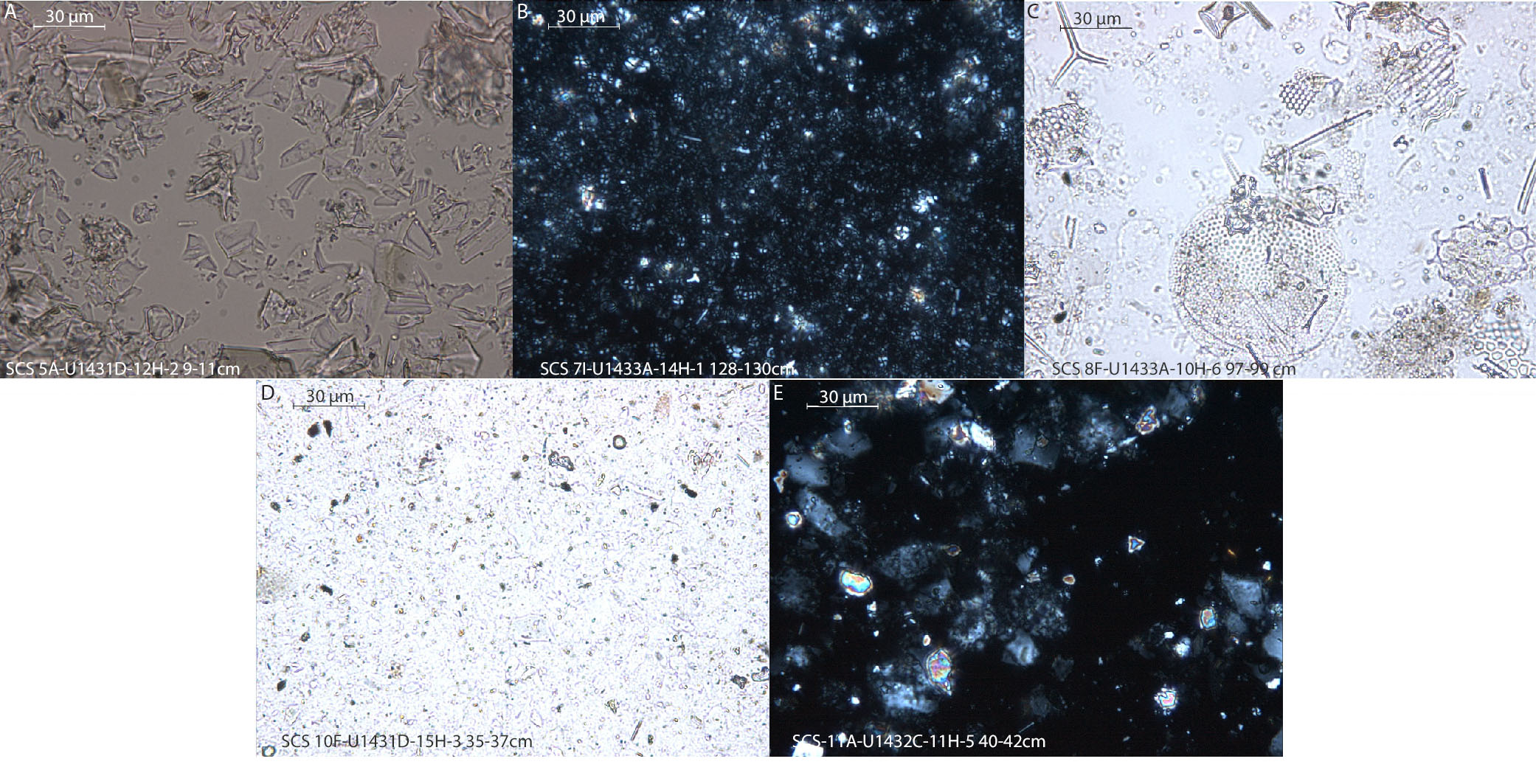


**Figure S4.** Rank-transformed Bray-Curtis dissimilarity between microbial communities sampled from adjacent sediment horizons of differing sediment types. The average dissimilarity between communities from adjacent sediment horizons was higher among communities in the methanogenic zone than among those in the sulfate reduction zone (Wilcoxon rank-sum *p* = 0.023).


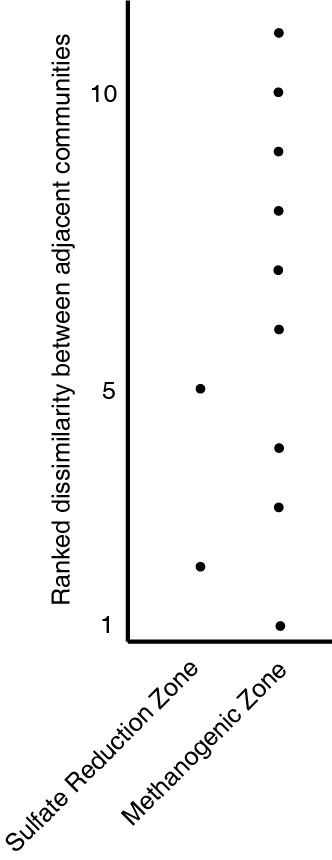

Supplement: Supplementary file 1 [file DataSheet1.docx]
